# Conservative Approach for the Management of Odontogenic Keratocyst: Our Experience

**DOI:** 10.7759/cureus.69306

**Published:** 2024-09-13

**Authors:** Rakshak Anand, Yajas Kumar, Nitin Bhagat

**Affiliations:** 1 Department of Oral and Maxillofacial Surgery, Manav Rachna Dental College, Faridabad, IND

**Keywords:** conservative management, enucleation, marsupialization, odontogenic keratocyst, recurrence

## Abstract

Odontogenic keratocyst (OKC) is defined as an intraosseous lesion of odontogenic origin, which can be unicystic or polycystic. The selection of a treatment strategy is complex, and several conservative and surgical approaches with varying rates of recurrence have been covered in the literature. To prevent further damage in these individuals, patients should have the proper diagnostic and treatment plan taken into consideration. For proliferative and osteolytic lesions in gnathic bones, OKC should be ruled out as a differential diagnosis. In this article, we present three cases who had OKC diagnoses and underwent enucleation; so far, there has been no recurrence.

## Introduction

Odontogenic keratocysts (OKCs) have long been known for their variegated origin and development, aggressive behavior, high recurrence, treatment modalities, association with Nevoid basal cell carcinoma syndrome (NBCC), transformation to an ameloblastoma and squamous cell carcinoma, and debatable nomenclature lately. They were titled keratocystic odontogenic tumor (KCOT) by the World Health Organization (WHO) [1] owing to their aggressive and recurrent nature. In 2017, WHO [2] put it back into the cystic category based on well-documented data that it regresses well following decompression. The conservative management of OKCs often involves decompression and marsupialization as preliminary steps to reduce the cyst size and intracystic pressure, thereby facilitating subsequent surgical interventions and minimizing recurrence rates. Decompression entails creating a small opening in the cyst to allow the continuous drainage of the cystic fluid, which leads to a reduction in the size of the cyst. This method is particularly advantageous for large cysts as it can alleviate symptoms and reduce the need for extensive surgical procedures. Following decompression, marsupialization may be done, where the cystic lining is sutured to the oral mucosa to keep the opening patent. This ensures ongoing drainage and a further reduction in cyst size, making the cyst more manageable for definitive treatments such as enucleation [3].

Enucleation is often regarded as the treatment of choice for OKCs due to its ability to completely remove the cyst and reduce the risk of recurrence. This surgical technique involves the full excision of the cystic lining and contents, thereby eliminating the source of the cyst and minimizing the potential for regrowth. The primary advantage of enucleation lies in its thoroughness, providing an immediate resolution to the cystic lesion and allowing for complete histopathological examination, which is crucial for confirming the diagnosis and ensuring that no malignant transformation has occurred [4].

## Case presentation

Case 1

**Figure 1:**
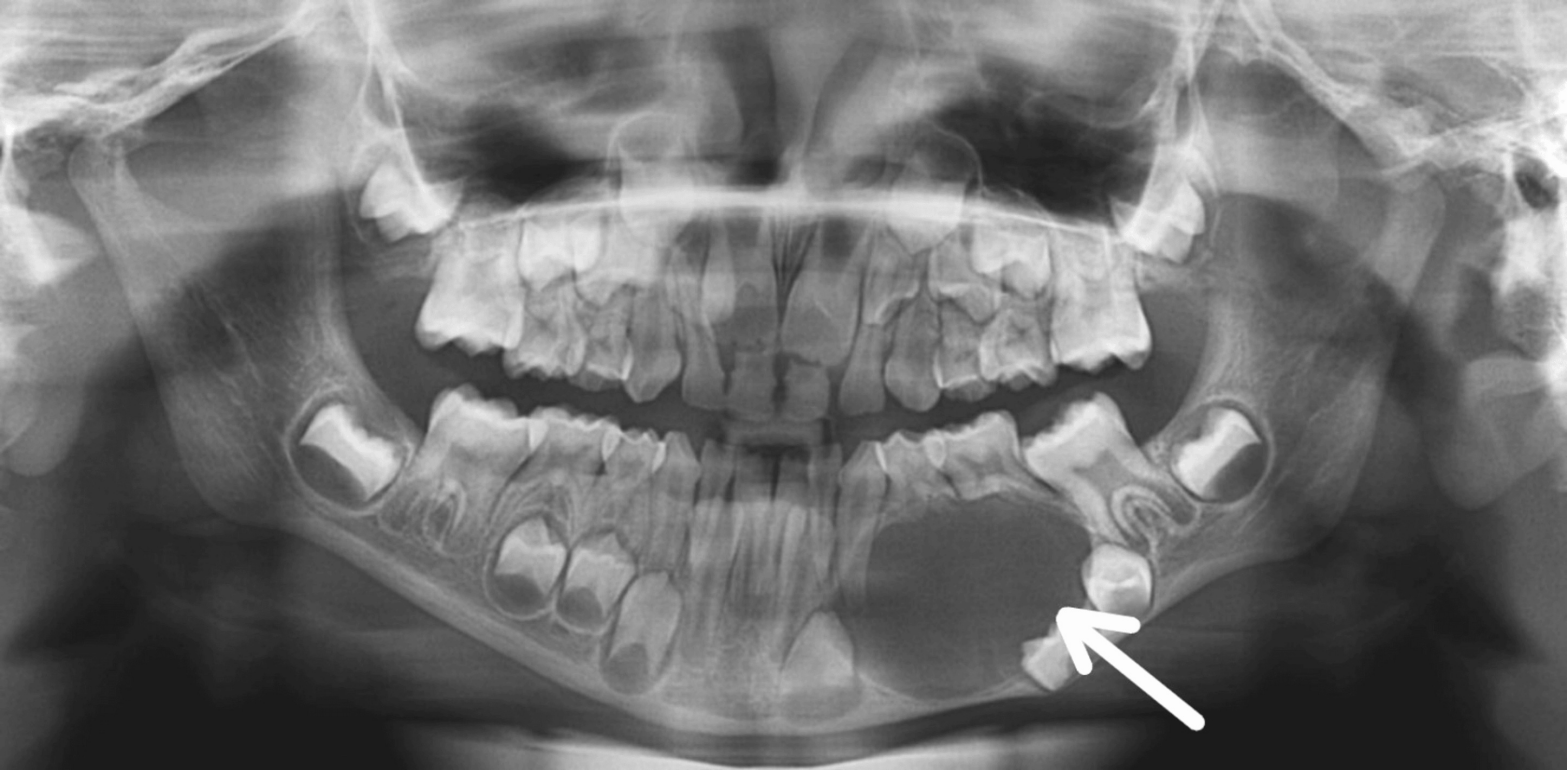
Preoperative radiograph showing unilocular radiolucency in the left posterior mandible involving 73, 74, and 75.

A six-year-old male patient reported to the outpatient department of Manav Rachna Dental College, Haryana, with a chief complaint of a hard, gradually increasing, painful swelling in the left mandibular premolar region, with no history of trauma. Anamnesis was non-contributory. Physical examination was done to rule out nevoid basal-cell carcinoma syndrome. Intraoral examination revealed a solitary, diffuse, bony hard, tender, swelling with intact mucosa present in the left mandibular premolar region, measuring approximately 2 x 1 cm in diameter. The associated teeth (74 and 75) were assigned Grade II mobility. Orthopantomogram (OPG) showed a smooth, unilocular radiolucency with corticated borders measuring 3 x 2.5 cms in regions 73, 74, and 75 with root resorption in relation to 74 and 75 along with unerupted 33, 34, and 35 (Figure 1).

**Figure 2:**
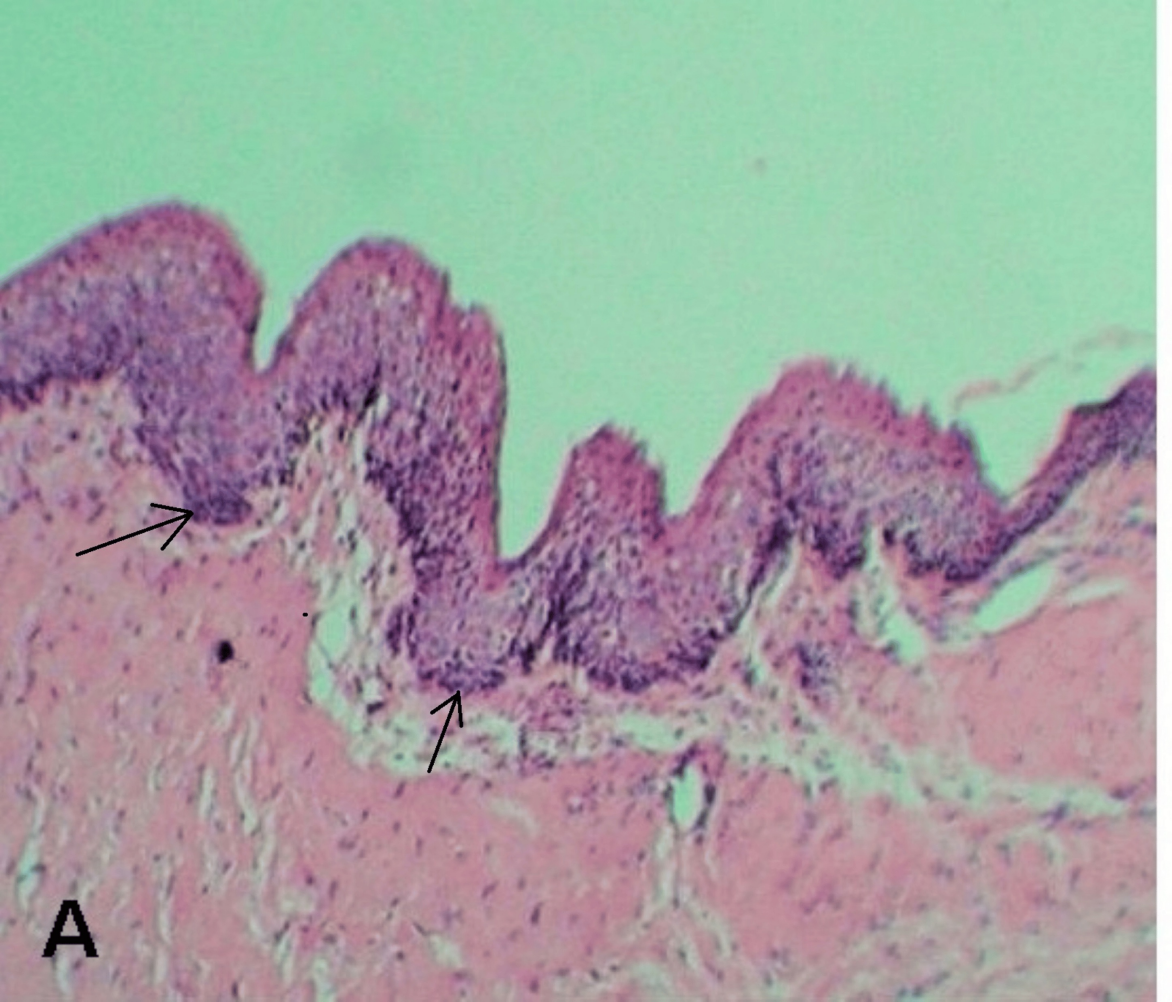
Hematoxylin and eosin staining 10X - Cystic lining showing a uniform para-keratinized epithelium.

Fine needle aspiration cytology (FNAC) revealed dirty yellowish, cheesy, viscoid material with total soluble protein content less than 4 gm/dL. Surgical management included incisional biopsy, after which the specimen was sent for histopathological examination (HPE). The H&E-stained tissue section revealed a cystic lumen containing abundant keratin. The lumen was lined by 5-8 cell thick corrugated parakeratinised stratified squamous epithelium exhibiting tall columnar cells with palisading nuclei. Based on these findings, a confirmed diagnosis of OKC was made (Figure 2).

**Figure 3:**
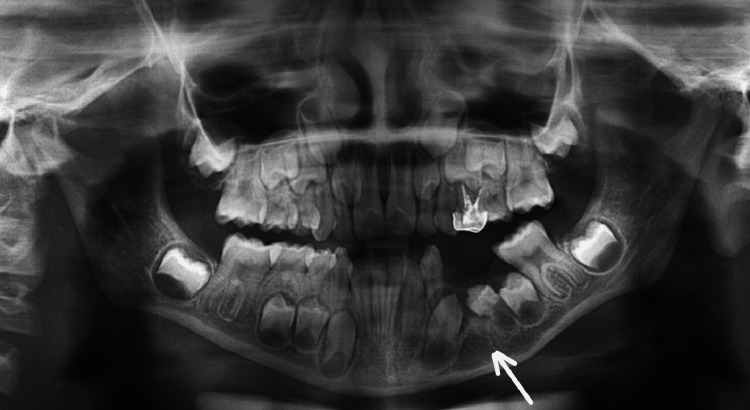
Postoperative radiograph showing no recurrence and bone formation.

An elective decision to operate on the patient under general anaesthesia was made. Under all aseptic conditions, local anesthesia with adrenaline in 1:200000 concentration was given as left lower inferior alveolar and lingual nerve block. The crevicular incision was made from 72 with a releasing incision anteriorly to 36 distally. Full thickness mucoperiosteal flap was raised, followed by extraction of 73, 74, and 75. The cystic lesion was then enucleated, and the site was closed primarily using a 4-0 absorbable surgical suture (braided coated polyglactin 910 violet). The subsequent OPGs show satisfactory bone formation with well-developing permanent teeth below (Figure 3).

We advocate the procedure has maintained a good quality life of for the patient, who is under a close follow-up with no recurrence for three years.

Case 2

**Figure 4:**
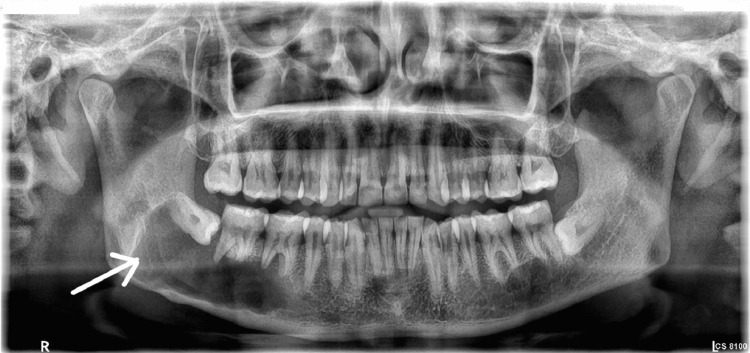
Preoperative panoramic radiograph showing a radiolucent lesion with respect to the right posterior mandible with an impacted right third molar.

A 36-year-old otherwise healthy male reported to the outpatient department of Manav Rachna Dental College, Haryana, with a chief complaint of hard, painful swelling in the right mandibular molar region. The patient gave no history of trauma or tobacco consumption. A history of loose teeth in the right lower jaw was reported by the patient for which he had sought no medical intervention. On examination, bony hard and tender swelling with buccolingual expansion was observed in relation to the right posterior mandible with mobility in associated teeth (46, 47). OPG showed a smooth, unilocular radiolucency with corticated borders measuring 7 x 2.5 cm in regions 46 and 47. Region 48 was found to be impacted and displaced (Figure 4).

**Figure 5:**
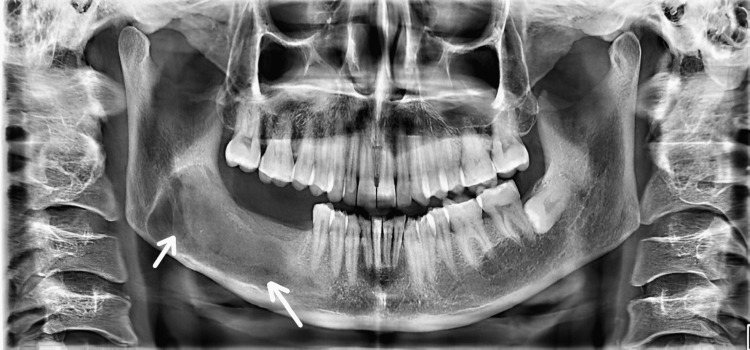
One-year postoperative follow-up radiograph showing no recurrence and bone formation.

Surgical management was initiated with informed consent and incisional biopsy; HPE revealed it to be an inflamed odontogenic keratocyst, and a plan of enucleation, followed by peripheral ostectomy, was made as the patient refused aggressive treatment. Under local anaesthesia, a full-thickness mucoperiosteal flap was reflected after giving a crevicular incision from 46 to 48 with an anterior releasing incision sparing the nervus mentalis and distal releasing incision overlying the external oblique ridge. Extraction was done with respect to 46, 47, and 48, along with enucleation of the cystic lesion. Peripheral ostectomy was carried out using rose bur, and the site was closed primarily using a 3-0 silk suture on a reverse cutting needle. The patient is now asymptomatic with regular follow-up for one and a half years with no recurrence to date (Figure 5).

Case 3

**Figure 6:**
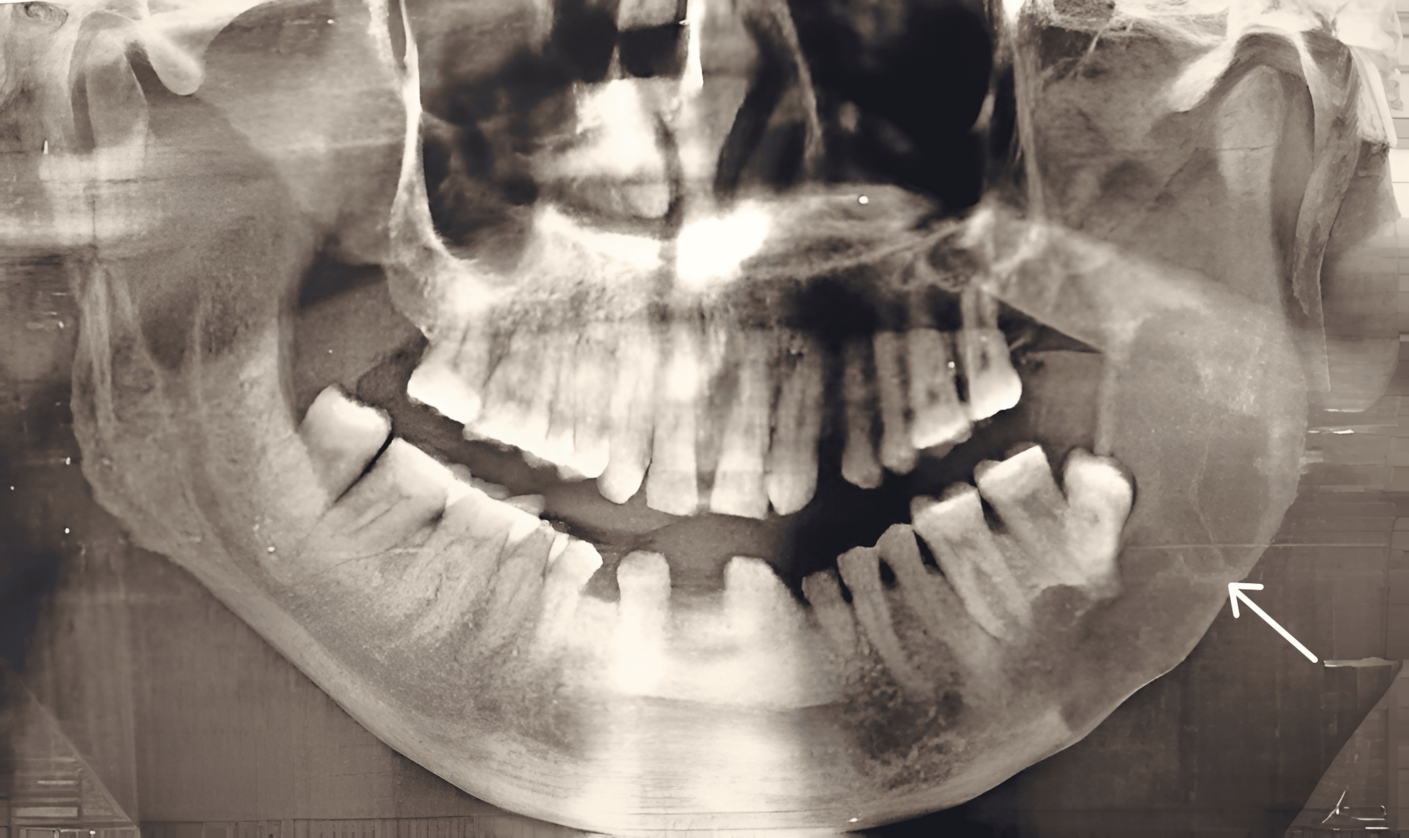
Preoperative radiograph showing a radiolucent lesion in the left posterior mandible.

A 50-year-old male reported to the outpatient department of Manav Rachna Dental College, Haryana, with a chief complaint of dull pain in the lower left back tooth region. The patient had no deleterious habits, and his oral hygiene status was adequate. General medical history revealed that the patient was suffering from type 2 diabetes mellitus and stage 1 hypertension. On examination, hard bony and tender swelling with buccolingual expansion was noted in the left posterior mandible with Grade I mobility of 35 and Grade II mobility with respect to 36 and 37. OPG showed a smooth, unilocular radiolucent lesion measuring up to 12 x 3.5 cm extending from 35 anteriorly to the ramus of the mandible posteriorly along. Region 38 seemed to be impacted and displaced (Figure 6).

**Figure 7:**
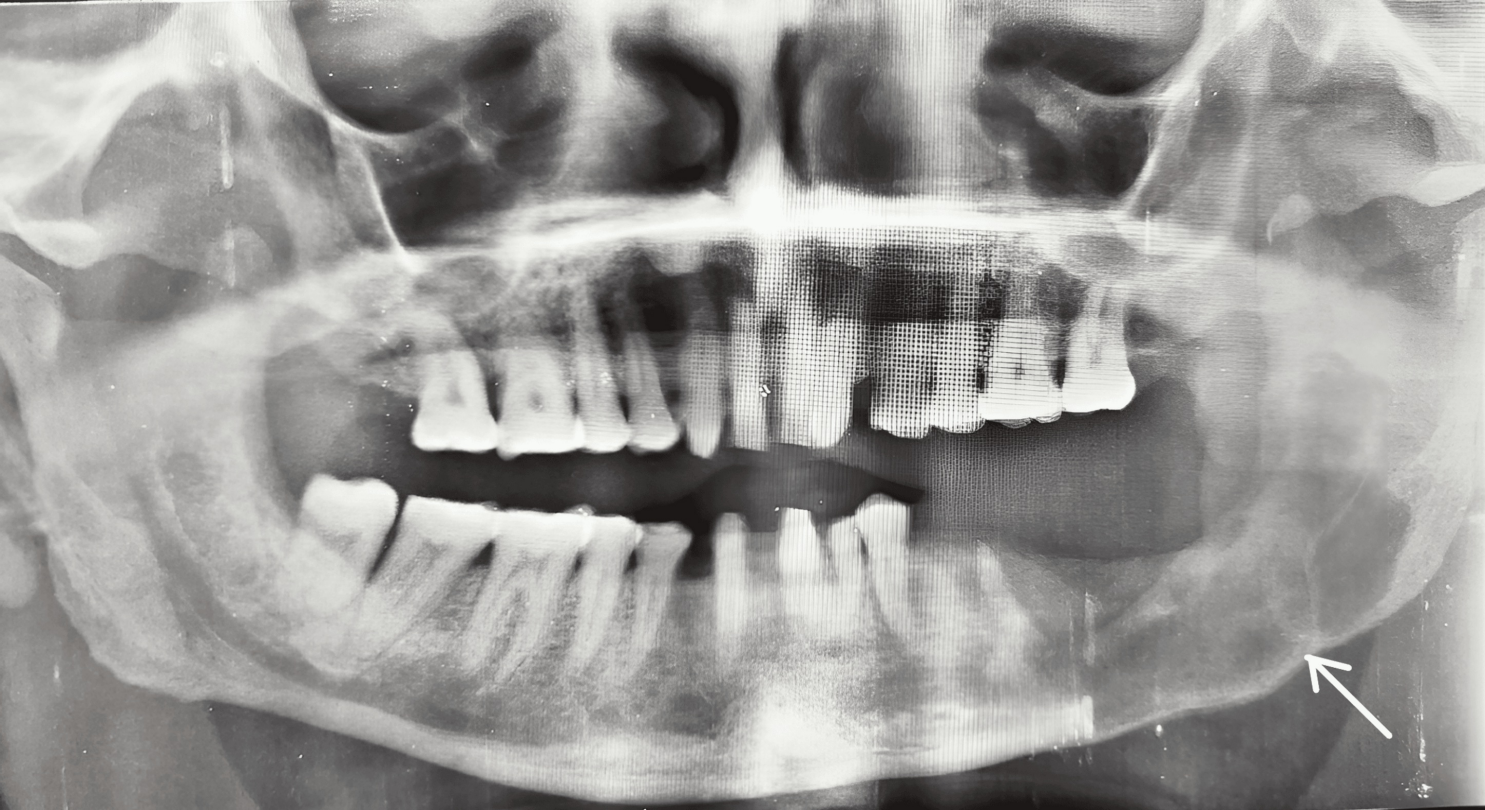
Six months postoperative follow-up radiograph showing no recurrence and initial bone formation.

FNAC of the lesion was negative, and initial suspicions of OKC were confirmed with incisional biopsy and HPE. A plan for extraction of the involved teeth and enucleation of the lesion was made as the patient had other comorbidities that did not permit repeated surgeries, neither was the patient willing for aggressive resection. Follow-up after six months revealed an intact lower border of the mandible and no recurrence (Figure 7).

The patient continues to be on close observation and follow-up.

## Discussion

OKC has been known in the literature for a long time. Philipsen [1] was the first to describe it in the year 1956. It is defined as “a benign uni- or multicystic, intraosseous tumor of odontogenic origin, with a characteristic lining of parakeratinized stratified squamous epithelium and potential for aggressive, infiltrative behaviour". The genetic basis for this was a tumor suppressor gene, PTCH (“patched”), which is involved in both sporadic KCOTs and NBCCS occurring on chromosome 9q22.3-q31, and if regular functioning of PTCH [5] vanishes, the proliferation-stimulating effects of SMO (smoothened) predominate. According to Chuong et al. [6], OKC comprises approximately 3-11% of odontogenic cysts. Mostly, they occur in the posterior mandible and radiographically present as unilocular or multilocular radiolucency. Multiple OKCs are a part of nevoid basal cell carcinoma syndrome (Gorlin-Goltz syndrome) [7]. They have been reported to transform into ameloblastoma and primary intraosseous squamous cell carcinoma (PIOSCC) [8]. Gang et al. [9] observed that they tend to grow in the anteroposterior direction without causing obvious bone expansion, which is why they are observed late by the patients. The recurrence rates of OKC [10] vary a lot ranging from 0% to 62.5% with the majority of cases showing recurrence in the first five years. As per Morgan et al. [11], a recurrence rate of 78% occurred in five years or less, while 22% occurred after five years of initial treatment. It presents mostly in the second to fourth decades of life (54.2%), but rare cases have been reported as early as the first decade and as late as the ninth decade of life [12].

The choice of treatment should be based on multiple factors such as the age of the patient, the clinical extent of the pathology, location and proximity to vital structures, mobility of overlying teeth, the radiographic appearance of the lesion, soft tissue involvement, variant of the lesion, presence of daughter cysts, and the recurrence rate. The various treatment modalities are debatable for their pros and cons. Conservative management includes simple enucleation, with or without curettage, or marsupialization. Aggressive treatment usually includes cryotherapy, electrocautery, peripheral ostectomy, or enbloc/segmental resection. However, the treatment of the OKC remains controversial. Nakamura et al. in their case series of 28 patients reported marsupialization as an effective treatment modality in treating large OKC with no recurrence [13]. de Molon et al. showed no recurrence in an OKC case treated by the same modality, with a follow-up period of five years [14].

In a case series of 23 patients, Marker et al. [15] concluded that decompression with and without enucleation is a successful modality with a low recurrence rate. Less than 1% of OKC cases occur in the maxilla with sinus involvement. Torres-Lagares et al. [16] and Ohki [17] advocated marsupialization as a successful treatment modality for OKC in the maxillary sinus. We also present a case series of nine individuals who were diagnosed with OKC; two of the cases include the maxillary sinus and a pediatric patient, respectively, and are not frequently seen. All the cases were treated successfully by enucleation and peripheral ostectomy with no recurrence to date with an average period of follow-up ranging from one to three years.

The technique of marsupialization, as described by Pogrel, is to make a window of at least a cm into the cyst, and the outline of the cyst mucosa is sutured with the oral mucosa. Pogrel [18,19] also stated that decompression relieves the pressure within the cystic cavity and thus augments new bone fill. It has been well-documented that, after decompression and marsupialization, the cyst lining undergoes histological changes and is eventually replaced by oral nonkeratinizing epithelium. The same was observed in our cases also.

As advocated by Stoelinga [20], the bony defect can be additionally treated with carnoy’s solution too to further limit the recurrence. Its average depth of penetration is 1.54 mm after five minutes of application. The accumulated evidence from studies of different treatments shows that resection of the odontogenic keratocyst is the most effective for the avoidance of recurrence. However, there is a general consensus in the literature that, in most cases, resection is an unnecessarily aggressive treatment for a benign cystic lesion. The most effective treatment therefore appears to be enucleation with or without peripheral ostectomy, followed by treatment with Carnoy’s solution. This may result in a recurrence rate of less than 2% [20].

This case report underscores the need for high-quality randomized controlled trials (RCTs) to establish effective treatment protocols for odontogenic keratocysts. Without such evidence, clinicians must rely on less robust studies, expert opinions, and clinical experience, which may lead to variability in treatment practices and outcomes.

## Conclusions

In the management of OKCs, we advocate for a conservative treatment approach that involves enucleation, followed by peripheral ostectomy. This strategy is particularly effective because it helps preserve both the functional integrity and aesthetic appearance of the affected areas in adult patients. Furthermore, it supports the normal growth potential of the jaw in pediatric patients, which is crucial for their overall development.

Although research indicates that, after a procedure such as marsupialization, the lining of an OKC transforms into normal oral epithelium once it is exposed to the oral cavity; this transition is beneficial as it reduces the potential for recurrence and promotes healing. Among the various treatment modalities available for OKCs, our primary objective is to select a method that not only minimizes the risk of recurrence but also results in the least amount of morbidity. This conservative approach, by focusing on enucleation and peripheral ostectomy, has consistently shown to be effective in managing OKCs with favorable outcomes. Through years of clinical practice and observation, conservative management of OKCs may prove its efficacy and reliability. It continues to be a preferred approach due to its balance of efficacy, minimal risk of recurrence, and reduced impact on patient quality of life.
